# King Edward's Hospital Fund for London

**Published:** 1912-12-21

**Authors:** 


### KING EDWARD'S HOSPITAL FUND FOR LONDON.

### The Speaker on a Diminishing Charitable Public.

The following is the full text of the speech delivered by the Speaker of the House of Commons in moving the adoption of the Reports and Awards at the meeting of the Governors and General Council of King Edward's Hospital Fund for London for the purpose of awarding grants, which was held at York House, St. James's Palace, on Wednesday, December 18:—

My Lords and Gentlemen,—His Highness the Duke of Teck has written to express his regret that he is unable to be present this morning, and on behalf of the Governors I rise, therefore, to move the adoption of the Reports of the Distribution and Convalescent Homes Committees.

### The King's Message.

Before doing so, however, I have the honour to read to you the following letter which has been addressed to the Governors by Lord Stamfordham, by direction of his Majesty the King, the Patron of the Fund:—

"Buckingham Palace,
"17th December, 1912.

"Dear Sir,—I am commanded by the King to convey to you, and through you to the Council at their meeting on Wednesday, an assurance of his Majesty's continued deep interest in the Fund.

"His Majesty is sorry to see the falling off in the income of the Fund from subscriptions and donations this year, but he is glad that, notwithstanding, it has been found possible to distribute, apart from Sir Ernest Cassel's munificent gift of last year, the same amount as in 1911. For the King would look upon any reduction in the total grants as a calamity, and is sure that this feeling would be shared by all those concerned, directly or indirectly, with the hospital work of London.

"His Majesty highly appreciates the work of the members of the Council and the Standing Committees, especially the Honorary Secretaries and Mr. Fry, and also the valuable services rendered by Lord Mersey, the Bishop of Stepney, and the late Lord Northcote in connection with the report on the out-patient question.

"His Majesty is gratified to hear of the excellent work of Mr. Maynard and the permanent staff.

"Yours very truly,
"Stamfordham.

"To the Presiding Governor of King Edward's Hospital Fund for London."

### The First Check.

The most important feature of the reports which are before us is the fact, referred to in his Majesty's gracious letter, that the amount of this year's distribution is the same as that of last year's—omitting, of course, from the comparison the supplementary grants by which the generosity of Sir Ernest Cassel enabled us to increase last year's awards. This is the first check in the steady rise in the amount distributed that has occurred since 1904. I need hardly say that the reason for the present pause is not to be found in any reduction of the needs of the hospitals. It is due solely to a falling off during the year in the income of the Fund. The income, which last year amounted to £184,000, is estimated to amount this year to a little over £134,000. It is therefore only by drawing on the surpluses of past fat years that a distribution of £157,500 has been possible in this lean year. But this is a method of making both ends meet which cannot go on, and it would have been imprudent to continue to increase the amount distributed in the face of so disquieting a diminution in the amount received.

### "A Steady Though Gradual Decline."

The diminution itself is shown in three important items in the receipts: Annual subscriptions, donations, and legacies. In the case of legacies there has been a falling off from £48,500 to £11,000. This, of course, accounts for the greater part of the reduction, and it shows how uncertain this source of income is. In the case of donations there is a drop of about £9,000. Last year, as in the year before, we were fortunate enough to receive an anonymous donation of £10,000. These generous gifts helped to justify us in increasing our previous distributions. But obviously, as a source of income, donations cannot be relied on, though our present position shows how extremely valuable a timely gift on this scale may be.

Annual subscriptions are in a different category; now it does seem to me that if the charitable public were to realise the demands upon the Fund and the great influence for good such a Fund can and, we believe, does exercise amongst the hospitals, the list of annual subscriptions would cease to show the decline which unfortunately it exhibits. The total is raised now and again by the receipt of munificent subscriptions such as those of Mr. Walter Morrison and the late Mrs. Lewis Hill, but apart from these there has been during the past a steady though gradual decline. Of course, we do not forget the amounts collected by the League of Mercy. But apart from the limited number of wealthy subscribers such as those I have mentioned, and the givers of small sums, whose valuable efforts the League of Mercy has organised with such devotion, there must be large classes who could give to hospitals, either directly or through the Fund, but who, for some reason or other, do not.

If any of those of whom I am speaking are under the impression that the King's Fund does not need their support—that it can go on growing automatically by means of legacies and income from investments—this year's record should surely dispel that illusion. The income from investments only provides about half of the annual distribution, and, even with all the skill of our Finance Committee, that income cannot be maintained if the falling off of other receipts compels the sale of securities. Donations and legacies are at best uncertain, and to ensure, therefore, the maintenance of our distribution, a steady income from annual subscribers is of urgent importance. (Hear, hear.)

**The Attitude of St. George's Hospital.**

Let us turn now from our income to our output, using the word in its widest sense to cover not only the grants but the whole work of the year. The distribution, though the same as last year in total amount, shows nevertheless variations in the detail of the separate awards which testify to the minute care devoted by the two distributing committees to the study of the relative claims of the very numerous institutions applying for grants. With regard to the renewed application from St. George's Hospital, I will only say this: We are bound, of course, by the special trusts attaching to the Fund, including the undertaking given by its founder, that the whole of it should go towards the relief of the sick poor and no part towards the support of medical education or research. But while we maintain the second part of this trust to the best of our abilities, we do not forget the first, and we view with the greatest regret any circumstance which removes from our list of grants, even for a time, any institution which does good work amongst the sick poor and is in need of money for that work. (Hear, hear.) We welcome, therefore, the fact that St. George's have been willing to reopen communications with the Fund, and have laid before us information as to their reasons for not accepting the conclusion of this Council upon the facts relating to their last application a year and a-half ago. (Hear, hear.)

In July last the Special Committee on the Out-patient question issued their report. I am sure you will be with me when I tender to Lord Mersey and the Bishop of Stepney our best thanks for the very valuable services they have rendered in undertaking this laborious inquiry and in preparing their exhaustive Report. Their findings and recommendations have been receiving the careful consideration of our Executive Committee, whose report on the subject will be laid before you this morning. One point that will, I feel sure, not be overlooked, is that the principal recommendations of the Committee are based upon reforms which are already in operation to a greater or less degree in many of the out-patient departments to-day, and involve, therefore, the extension of tried remedies rather than the introduction of new and revolutionary methods. But even such an extension must be a matter of time and of the careful adjustment of general principles to particular circumstances, and this is especially the case at the present moment, when great changes in connection with the treatment of a large proportion of the sick poor are imminent.

How the Fund Assists Economy.

It is now two years since the Fund's Annual Statistical Report on the expenditure of the London Hospitals reached its present form with the inclusion of all the hospitals whose accounts are comparable. By the annual publication of these comparative figures the Fund continues to exercise its influence in the direction of economy and to furnish the hospitals with information which may assist them in their own efforts. It is a remarkable fact that in spite of the continual introduction of more costly methods of medical treatment, and the steady rise in general prices, the hospitals have achieved, since the first issue of these reports, savings calculated at no less than £37,000 a year. (Hear, hear.)

We have this year to lament the loss by death of three members of the Council. Dr. Bowman Stephenson and Sir Julius Wernher had both been members since the foundation of the Fund in 1897, while Sir Joseph Dimsdale joined in 1901. We have also lost in Sir William Allchin, Mr. Clinton Dent, and Mr. Willie James three visitors who had all done valuable service for several years.

It now remains for me to tender to the members of the Council, and members of committees, and visitors, and honorary officers the best thanks of the governors for all the self-sacrificing labours upon which the welfare of the Fund so largely depends. We are particularly indebted, I think, to the Honorary Secretaries, who have had a busy year, and to Mr. Fry, who very kindly continued, in connection with the Out-patient Inquiry, the work which he had begun when himself an Honorary Secretary. (Hear, hear.)

I now, therefore, formally beg to move the adoption of the Reports of the Distribution and Convalescent Homes Committees.

Lord Stamfordham having seconded the motion, the adoption of the reports was then put and carried unanimously.

